# CYPstrate: A Set of Machine Learning Models for the Accurate Classification of Cytochrome P450 Enzyme Substrates and Non-Substrates

**DOI:** 10.3390/molecules26154678

**Published:** 2021-08-02

**Authors:** Malte Holmer, Christina de Bruyn Kops, Conrad Stork, Johannes Kirchmair

**Affiliations:** 1 Center for Bioinformatics (ZBH), Department of Informatics, Universität Hamburg, 20146 Hamburg, Germany; malte.holmer@studium.uni-hamburg.de (M.H.); kops@zbh.uni-hamburg.de (C.d.B.K.); stork@zbh.uni-hamburg.de (C.S.); 2 Division of Pharmaceutical Chemistry, Department of Pharmaceutical Sciences, University of Vienna, 1090 Vienna, Austria

**Keywords:** drug metabolism prediction, cytochrome P450 enzymes, substrates, machine learning, classification

## Abstract

The interaction of small organic molecules such as drugs, agrochemicals, and cosmetics with cytochrome P450 enzymes (CYPs) can lead to substantial changes in the bioavailability of active substances and hence consequences with respect to pharmacological efficacy and toxicity. Therefore, efficient means of predicting the interactions of small organic molecules with CYPs are of high importance to a host of different industries. In this work, we present a new set of machine learning models for the classification of xenobiotics into substrates and non-substrates of nine human CYP isozymes: CYPs 1A2, 2A6, 2B6, 2C8, 2C9, 2C19, 2D6, 2E1, and 3A4. The models are trained on an extended, high-quality collection of known substrates and non-substrates and have been subjected to thorough validation. Our results show that the models yield competitive performance and are favorable for the detection of CYP substrates. In particular, a new consensus model reached high performance, with Matthews correlation coefficients (MCCs) between 0.45 (CYP2C8) and 0.85 (CYP3A4), although at the cost of coverage. The best models presented in this work are accessible free of charge via the “CYPstrate” module of the New E-Resource for Drug Discovery (NERDD).

## 1. Introduction

The cytochrome P450 (CYP) family of enzymes metabolizes a wide range of xenobiotics. In particular CYPs 1A2, 2A6, 2B6, 2C8, 2C9, 2C19, 2D6, 3A4, and 2E1 are of relevance to the metabolism of drugs [1], natural products, agrochemicals, and cosmetics [2,3,4]. Metabolism can lead to a gain or loss of biological activity, and in particular, CYP-mediated metabolism is linked to the formation of toxic (i.e., primarily reactive) metabolites and the causation of drug–drug interactions [3]. Therefore, the ability to predict the metabolic fate of small organic molecules is of high importance to a host of different industries [5].

Modern biochemical, cell-based, and analytical methods allow the determination of metabolism at an unprecedented level of detail, but they require significant resources, expertise, and time. In recent years, in particular machine learning methods [6,7,8,9] have seen significant progress and are increasingly becoming recognized as an important pillar of xenobiotic metabolism prediction.

One of the most active research areas in computational metabolism prediction has been the development of machine learning models for the prediction of the interactions of small molecules with CYP enzymes. As a result of these efforts, several models for the identification of CYP inhibitors and a few models for the identification of CYP substrates are at our disposal today (see ref [9] for a recent review).

Focusing on CYP substrate prediction, most of the available machine learning models are embedded in commercial platforms (e.g., ADMET Predictor [10], ADMEWORKS Predictor [11], the Percepta platform [12], and the WhichP450 [13] of StarDrop [14]). Several academic groups have reported the development of models for CYP substrate prediction, but most of these models are either not accessible or clearly limited in their quality or the quality of their documentation. From our careful review of the literature, we conclude that CypReact [15] is currently the best freely available model for CYP substrate prediction with respect to accuracy, coverage, and documentation. 

CypReact consists of a set of machine learning models, one for each of the nine CYPs covered by the tool (i.e., CYPs 1A2, 2A6, 2B6, 2C8, 2C9, 2C19, 2D6, 2E1, and 3A4). The models were selected by the systematic assessment of the performance of a variety of machine learning algorithms (i.e., support vector machines (SVMs), logistic regression, decision tree, random forest (RF), and an ensemble method) trained on physicochemical descriptors and molecular fingerprints. For the development and testing of the CypReact models, the authors utilized a well-known set of 679 experimentally confirmed substrates [16] and a set of 1053 non-substrates compiled from different sources [17,18,19,20]. The latter set of molecules was constructed to include compounds that are structurally related to the known substrates and hence pose a challenge to classifiers. During five-fold cross-validation (CV), the best models (which are based on 2279 features, including physicochemical descriptors and molecular fingerprints) achieved Jaccard scores between 0.25 and 0.55 for the different CYPs. CypReact was also evaluated on a test set, but the validity of these results is questionable because of some data leakage that may have occurred (see Section 2.6).

In this work, we present a new set of machine learning models for the classification of substrates and non-substrates for the nine above-mentioned human CYP isozymes. The models are trained on extended sets of high-quality data and subjected to thorough validation on holdout data. All of the best models are available via a free, public web service which, among several other features, provides an indicator of the reliability of the individual predictions. Furthermore, all training and test data utilized in this work are provided as Supporting Information.

## 2. Results and Discussion

### 2.1. Characterization of Data Sets

For the purpose of model development, a core data set consisting of 1831 compounds, both substrates and non-substrates, of nine human CYPs (1A2, 2A6, 2B6, 2C8, 2C9, 2C19, 2D6, 2E1, and 3A4) was compiled from the works of Tian et al. [15] and Hunt et al. [13]. Depending on the availability of bioactivity data in the data sets of Tian et al. and Hunt et al., binary activity labels (i.e., “substrate”, “non-substrate”) were assigned to all compounds for all CYPs. A compound deduplication routine was subsequently executed that checks for the consistency of the bioactivity labels assigned to one and the same molecule (while disregarding stereochemical information; see Section 3 Materials and Methods for details). This resulted in a high-quality, 94% complete bioactivity data matrix.

The number of substrates and non-substrates in the core data set is imbalanced for most CYPs, with the most pronounced imbalance observed for CYP2A6 (ratio of substrates to non-substrates 1:16). Importantly, the integration of the data set of Hunt et al. (which is exclusively composed of CYP substrates) led to the addition of 15 substrates on average (Table 1).

#### Analysis of the Relevance of the Core Data Set to Small-Molecule Research

**Figure 1:**
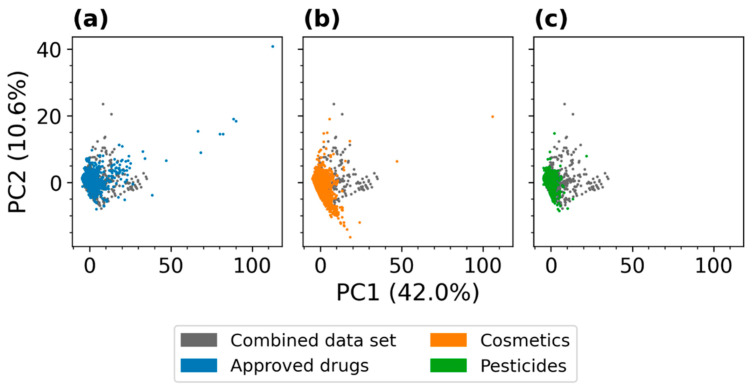
Principal component analysis (PCA) scatter plots comparing the chemical space of the core data set with that of the reference data sets of (**a**) approved drugs, (**b**) cosmetic ingredients, and (**c**) pesticides. The percentages reported in the axes labels denote the proportion of variance explained by the respective principal component. The principal components are calculated from 44 physically meaningful 2D molecular descriptors selected previously [23].

In order to understand the relevance of the core data set to small-molecule research, we conducted a comparative analysis of chemical space coverage for the core data set, the approved drugs subset of DrugBank [19], the cosmetic ingredients data set from COSMOS DB [21], and the set of agrochemicals from the Environmental Protection Agency’s (EPA’s) Pesticide Chemical Search Database [22]. The principal component analysis (PCA) scatter plots in Figure 1 show that the 1831 compounds of the core data set cover the chemical spaces of drugs, cosmetic ingredients, and agrochemicals quite well. Only a few compounds of the reference sets are not embedded in the point cloud formed by the compounds of the core data set. These outliers are mostly esters (with long, aliphatic chains), polypeptides, and polysaccharides.

**Figure 2:**
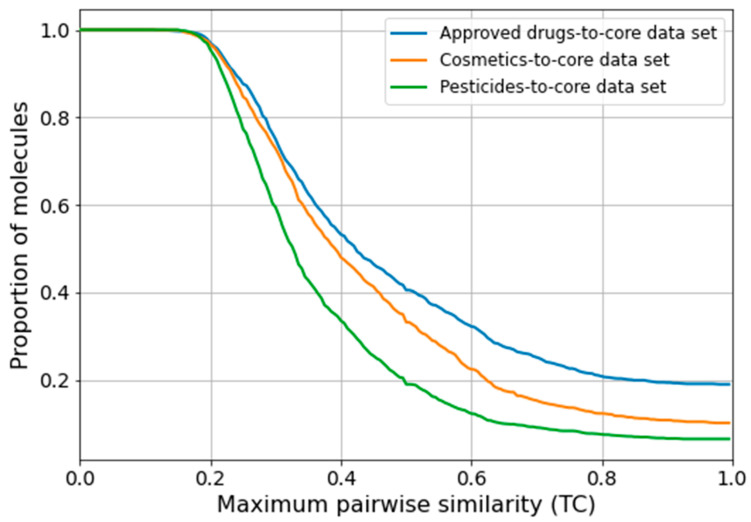
Proportion of molecules in the reference data sets (approved drugs, cosmetic ingredients, and pesticides) with a maximum pairwise molecular similarity above a certain threshold to the core data set. Similarity is expressed as the Tanimoto coefficient based on Morgan fingerprints (2048 bits, radius = 2).

In addition to PCA, we analyzed the pairwise maximum similarities (based on the Tanimoto coefficient derived from Morgan fingerprints with a length of 2048 bits and a radius of 2) between the molecules in each reference data set and the core data set. The proportions of molecules in each reference data set with a maximum pairwise similarity above a certain threshold are shown in Figure 2. For example, approximately 32% of the approved drugs have a Tanimoto coefficient of at least 0.6 to their nearest neighbor in the core data set, indicating substantial molecular similarity. In comparison, cosmetic ingredients and pesticides are less similar to the compounds in the core data set, overall. Considering the same Tanimoto coefficient (0.6), only 22% of the cosmetic ingredients and 12% of the pesticides are represented by the core data set.

### 2.2. Model Development

**Figure 3:**
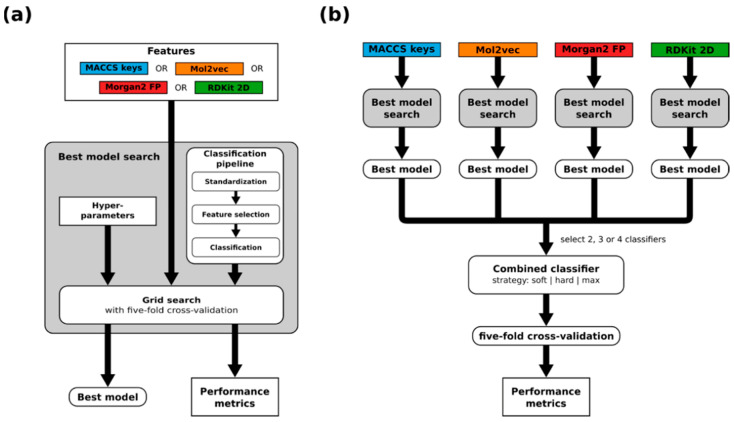
Overview of the modeling workflows utilized in this work. (**a**) Workflow for the generation of single models. (**b**) Workflow for the generation of combined models.

Random forest models and support vector machines for binary classification were trained individually for each of the nine CYPs. Model generation followed a consistent workflow (Figure 3a) that involves feature selection and, in the case of SVMs, the standardization of non-binary features. To address the imbalance in the data, balanced class weights were used for all models.

Prior to model development, the core data set was split into CYP isozyme-specific subsets, and any entries with missing activity labels were removed. Then, within each subset, the remaining compounds were assigned to a training set (80%) and a test set (20%) by stratified shuffled splitting. The total number of substrates and non-substrates in each of the data sets are listed in Table S1.

Any model described in the following sections has been trained on one CYP isozyme-specific training set and its hyperparameters have been optimized by grid search within a five-fold CV framework (see Section 3 Materials and Methods for details).

### 2.3. Performance of Single Classifiers

#### 2.3.1. Performance during Cross-Validation

**Figure 4:**
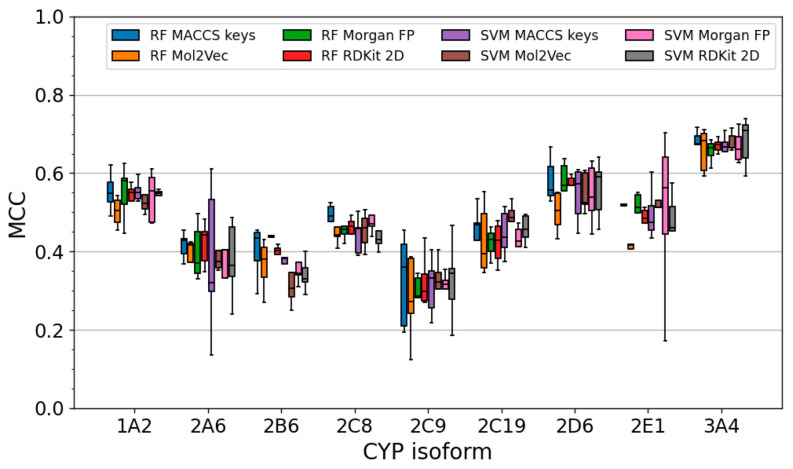
Five-fold CV performance (quantified as Matthews correlation coefficient, MCC) for all classifiers trained on a single type of feature (i.e., MACCS keys, Mol2vec descriptors, Morgan fingerprints, or RDKit 2D descriptors). Whiskers mark the range of the non-outlier data (defined as any data between lower quartile −1.5 interquartile range and upper quartile +1.5 interquartile range).

For each of the nine CYPs, eight individual classifiers were generated by combining two machine learning algorithms (RF and SVM) with four different feature sets (MACCS keys, Mol2vec descriptors, Morgan fingerprints, and RDKit [24] 2D descriptors). The performance of the classifiers was evaluated within a five-fold CV framework. As shown in Figure 4, the five-fold CV performance, evaluated by the Matthews correlation coefficient [25] (MCC), is comparable across the different types of models (i.e., different combinations of algorithms and feature sets). No particular combination of machine learning algorithm and descriptor set consistently outperformed the others. Differences in the performance of the individual models were particularly small for CYP3A4 (the maximum difference between the median MCC of the best-performing model and the worst one was just 0.05), followed by CYPs 1A2, 2C8, 2C9, 2C19, and 2D6 (up to 0.09). For CYPs 2A6, 2B6, and 2E1, somewhat larger differences were observed (up to 0.15). The best models for each CYP isozyme (in terms of MCC) are henceforth referred to as the “selected models”. Their performance data are reported in Table 2.

In contrast to the minor differences in performance observed for the various types of models, clear differences were recorded across the different CYPs. For the selected models, the highest median MCC during five-fold CV (0.71) was obtained for CYP3A4, whereas the worst median MCC (0.36) was obtained for CYP2C8 (Table 2). This divergence across the nine CYPs is likely a result of the limited sizes of the CYP-specific training sets and, in particular, substantial differences in the number of substrates included in respective training sets (e.g., 416 substrates for CYP3A4 versus 119 substrates for CYP2C8; see Table S1). Since the CYP3A4 classifier is trained on more positive samples than the classifiers for the other isozymes, better performance on substrates is expected. 

To test whether a correlation exists between model performance and the number of substrates in the training set, we calculated Spearman’s rho correlation coefficient (r_s_) between the median MCC obtained during CV and the number of substrates in the training set. For the 72 classifiers based on a single feature set, r_s_ was 0.86, indicating a strong correlation between the two variables (see Figure S1 for the scatter plot).

The selected models also performed well in compound ranking, which is relevant to applications such as virtual screening (Table 2). The area under the receiver operating characteristic curve (AUC) was between 0.80 (CYP2C8) and 0.92 (CYP3A4 and CYP2D6).

#### 2.3.2. Performance on the Test Set

In addition to CV, the selected models were evaluated on the test set, on which the models showed similar performance in terms of the MCC (ΔMCC −0.04, averaged over the 5 folds; Table 2). The highest increase in MCC on the test set (as compared to CV performance) was recorded for CYP2C19 (ΔMCC +0.10), and the strongest decrease was observed for CYP2E1 (ΔMCC −0.16).

#### 2.3.3. Performance Dependence on the Level of Structural Similarity during Cross-Validation

**Figure 5:**
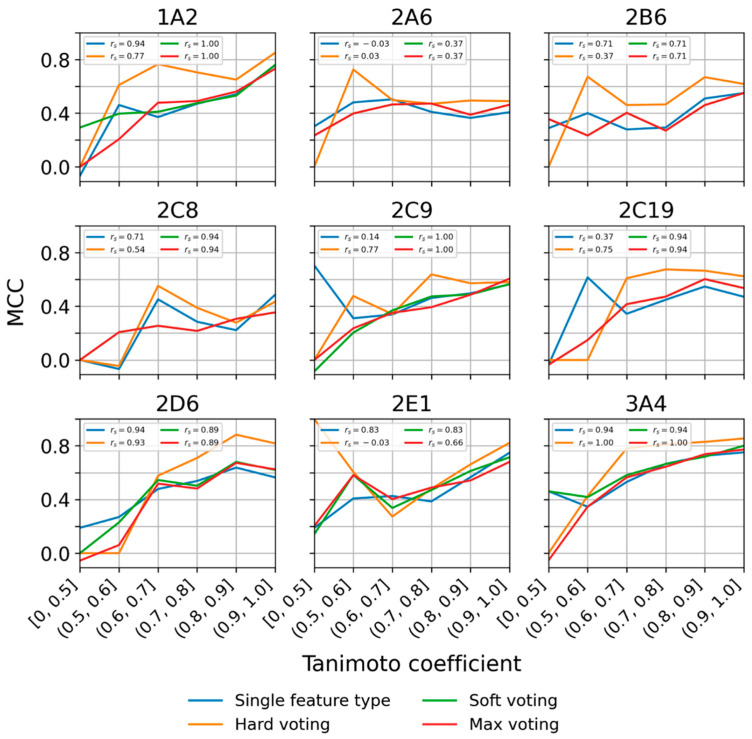
Model performance (quantified as MCC) as a function of molecular similarity between the compounds of the validation set and the nearest neighbor in the training set (quantified by the Tanimoto coefficient based on MACCS keys). Spearman’s rho values are reported in the figure legends.

In general, the likelihood of a prediction to be correct increases with the molecular similarity of the query molecule to the instances in the training data. This relationship holds true also for most of the selected models (we quantify molecular similarity of the query to the training data as the highest Tanimoto coefficient, based on Morgan2 fingerprints, between the test compound and any instance in the CYP-specific training set). For CYPs 1A2, 2B6, 2C8, 2D6, 2E1, and 3A4 r_s_ was between 0.71 and 0.94 (Figure 5). For CYPs 2A6, 2C9, and 2C19, the calculated coefficients did not indicate a clear correlation (r_s_ between −0.03 and 0.37), which is likely an artifact resulting from the limited data. Averaged over the nine selected models, the MCC was 0.51 for compounds represented by structurally related training instances (Tanimoto coefficient 0.7 or higher) and 0.23 for compounds more dissimilar to the training data (Tanimoto coefficient 0.5 or lower).

### 2.4. Analysis of Discriminative Features in Substrates and Non-Substrates

In order to obtain a better understanding of what structural patterns and physicochemical properties are important for the classifiers to recognize a compound as a substrate or non-substrate of a particular CYP, we analyzed the importance of the individual MACCS keys and RDKit 2D descriptors. This analysis is based on the Gini importance values assigned by the best-performing RF classifiers for each CYP (by MCC; classifiers based on a single set of features). Note that features removed by the variance or ANOVA-F score filter as part of the classification workflow are not considered in this analysis (see Section 3 Materials and Methods for details).

**Figure 6:**
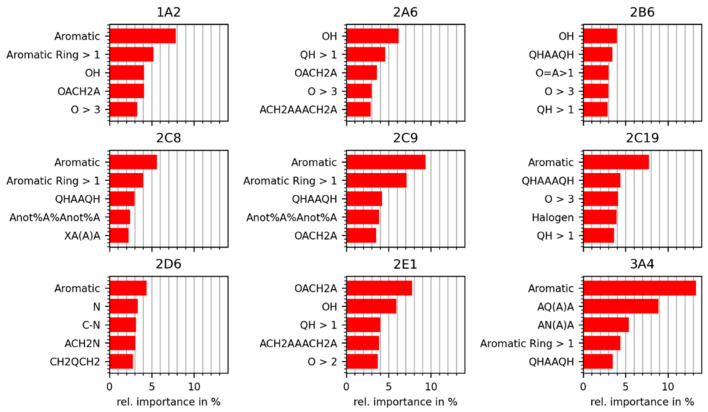
Relative feature importance values (calculated as Gini importance) of the five most relevant MACCS keys identified during 5-fold CV by the best RF classifiers (by MCC) trained on MACCS keys. In the reported patterns, “A” denotes any atom, “Q” denotes an atom that is not carbon or hydrogen, “X” denotes any halogen atom, “%” denotes an aromatic bond, and “not%” denotes a bond that is non-aromatic [29].

**Figure 7:**
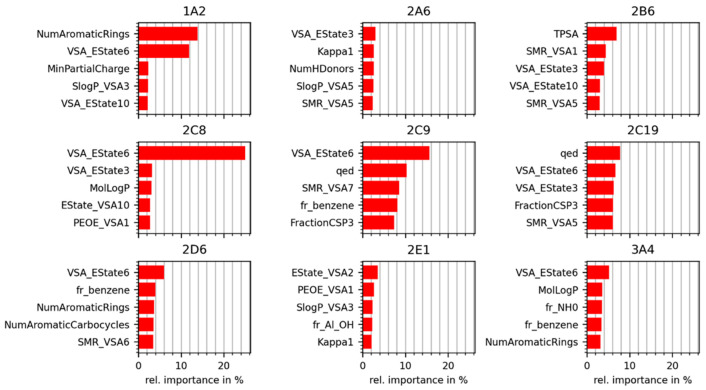
Relative feature importances (calculated as Gini importances) of the five most relevant RDKit 2D descriptors identified during 5-fold CV by the best RF classifiers (by MCC) trained on RDKit 2D descriptors. A detailed description of the descriptors is provided in Table S2.

The results of this analysis are described in the following subsections, and Figure 6 and Figure 7 provide a graphical overview of the importance of the five top-ranked MACCS keys and RDKit 2D descriptors, respectively. Note that high values for the relative importance of a feature can indicate the importance of either the presence or absence of a feature for a compound to be recognized as substrate of a certain CYP.

#### 2.4.1. Importance Calculated for Individual MACCS Keys

The aromaticity of compounds is clearly the most important molecular property for the discrimination of substrates and non-substrates: In six out of the nine investigated CYPs the most important MACCS feature for distinguishing substrates and non-substrates was “Aromatic”, which describes the presence of at least one aromatic atom in a molecule (Figure 6). In addition, the feature “Aromatic ring > 1” was ranked among the top five descriptors for four CYPs. It is well known that the aromaticity of small molecules is a decisive factor in their binding to various CYP isozymes [26,27,28].

The second discriminative property is the presence or absence of heteroatoms. Among the top-ranked MACCS keys are “OACH2A”, “O > 3”, and “OH”, all of which encode the presence or absence of oxygen atoms (“O” denotes an oxygen atom, “A” denotes any atom, and “CH2” denotes a methylene group), as well as “QHAAQH” and “QH > 1”, which encode moieties containing any heteroatoms (“Q”).

#### 2.4.2. Importances Calculated for Individual RDKit 2D Descriptors

The observations made on the importance of individual MACCS keys are largely consistent with those made for the individual RDKit 2D descriptors (Figure 7). Among the top-ranked features are several hybrid descriptors known from the Molecular Operating Environment (MOE) [30] that combine approximations of the van der Waals surface area (VSA) with properties such as electrotopological states (EState indices) [31], atom partial charges, molecular refractivity (SMR), or SlogP. The hybrid descriptors and others, including “fr_NH0” (number of tertiary amines), “NumHDonors” (number of hydrogen bond donors), and “fr Al OH” (fraction of aliphatic hydroxyl groups), are linked, to different extents, to the content of nitrogen and oxygen atoms in a molecule, to polarity and molecular topology.

Aromaticity is again clearly of relevance to classification. This is reflected by the high ranking of descriptors such “NumAromaticRings” (number of aromatic rings), “fr_benzene” (number of benzene rings, i.e., benzene ring fragments), and “NumAromaticCarbocycles” (number of aromatic carbocycles).

### 2.5. Performance of Combined Models

Considering only the best model (by MCC) of each combination of machine learning algorithm and feature set, we constructed models that combine two or more of these classifiers (Figure 3b). Three approaches to combine the predictions from individual classifiers were explored:

1. **Soft voting**, where the class probabilities reported by the individual models are averaged.
2. **Hard voting**, where the classifications of the individual classifiers are combined by identifying the consensus of the individual binary predictions. Importantly, hard voting does not produce predictions in some cases, because we require consensus models composed of two or three single classifiers to agree in their classifications in order to produce a prediction. For consensus models composed of four models, we tested two scenarios: one in which all models are required to agree and one in which at least three models are required to agree in order to produce a prediction.
3. **Max voting**, where the maximum class probability reported by any of the classifiers (taking into account both classes) is used for making a binary prediction. The maximum class probability is determined considering the class probabilities for both the substrate and non-substrate classes.

A total of 232 combined models were generated and tested, as presented in Table 3. From these 232 combined models, we identified the best combined model for each modeling strategy.

#### 2.5.1. Performance of Soft Voting Classifiers

**Figure 8:**
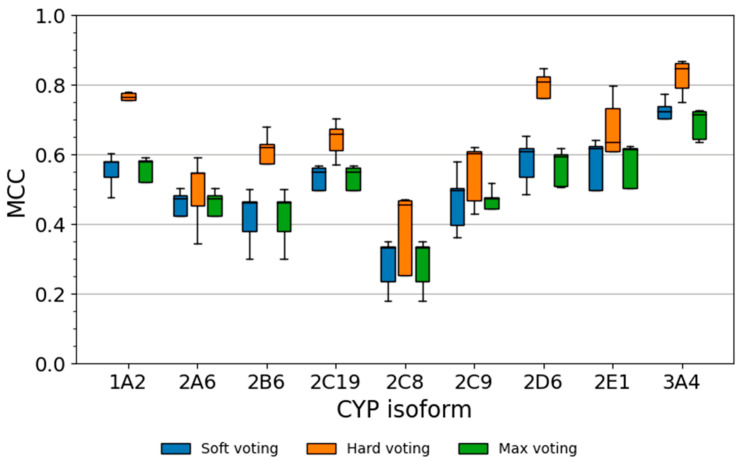
Five-fold CV performance (quantified as MCC) for the best combined models for each voting strategy. Whiskers mark the range of the non-outlier data which is defined as lower quartile −1.5 interquartile range up to upper quartile +1.5 interquartile range.

The soft classifiers showed good classification performance (MCCs up to 0.72 during CV and up to 0.70 on the test set; Figure 8 and Table 4) and ranking performance (AUC values up to 0.94 during CV and up to 0.92 on the test set), but they did not consistently outperform the single classifiers. For this reason, they were not further pursued.

#### 2.5.2. Performance of Hard Voting Classifiers

**Figure 9:**
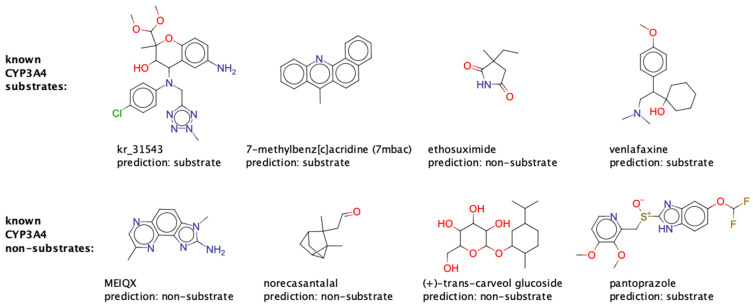
Eight representative examples of substrates and non-substrates included in the test set for CYP3A4 and how they are classified by the hard voting model.

The best hard voting classifiers for each of the CYP isozymes obtained superior prediction performance in comparison to the respective best single classifier (Figure 8 and Table 5). During CV, the median MCCs of the hard voting classifiers were between 0.45 (CYP2C8) and 0.85 (CYP3A4; Table 5). Compared to the respective single classifiers, this represents an increase in MCC by 0.07 (CYP2E1) to 0.22 (CYP2D6). Likewise, on the test set, the models obtained MCCs between 0.37 (CYP2A6) and 0.81 (CYP1A2 and CYP3A4), which correspond to improvements in MCC by +0.05 (CYP2A6) to +0.31 (CYP1A2) over the single classifier. The highest increase in MCC on the test set (as compared to CV performance) was recorded for CYP2C19 (ΔMCC +0.06), and the strongest decrease was observed for CYP2A6 (ΔMCC −0.18). Figure 9 shows predictions of the CYP3A4 hard voting classifier for eight representative examples of substrates and non-substrates included in this test set.

The fact that hard voting classifiers do not produce predictions for some molecules needs to be considered. In the CV experiments, the coverage of substrates was between 0.42 (CYP2C9) and 0.71 (CYP2A6; Table 5), and the coverage of non-substrates was between 0.79 (CYP2C9) and 0.93 (CYP2E1). On the test set, the coverage of substrates was between 0.39 (CYP2C9) and 0.72 (CYP3A4), whereas the coverage of non-substrates was between 0.80 (CYP2C9) and 0.92 (CYP3A4).

For most hard voting classifiers, a clear correlation between the similarity of queries to the training data and the prediction performance during CV was observed (Figure 5). For models for CYPs 1A2, 2C19, 2C8, 2C9, and 3A4, Spearman’s rho was between 0.54 and 1.0. For CYPs 2A6 and 2E1, no such correlation was observed (r_s_ = 0.03 and r_s_ = −0.03, respectively), which is likely related to the limited amount of available data (in particular on substrates). Averaged over the nine selected models, the MCC was 0.64 for compounds represented by structurally related training instances (Tanimoto coefficient 0.7 or higher) and 0.11 for compounds more dissimilar to the training data (Tanimoto coefficient 0.5 or lower).

#### 2.5.3. Performance of Max Voting Classifiers

In analogy to the observations made for the soft voting classifiers, no clear advantage of the max voting classifiers over the single models were identified. The max voting classifiers obtained MCCs up to 0.71 during CV and up to 0.71 on the test set (Table 6). Their AUC values were 0.92 for both CV and the test set. Therefore, the max voting classifiers were not further pursued.

### 2.6. Comparison of Our Models to CypReact

As mentioned in the Introduction, the CypReact collection of classifiers for the nine relevant CYP isozymes is the best free model for CYP substrate prediction available to date.

#### 2.6.1. Comparison of the Data Sets Employed for Model Training and Testing

From a data set perspective, the added value of our core data set compared to the CypReact data set is that it contains high-quality data on an additional 84 CYP substrates from the work of Hunt et al. Nevertheless, our CYP-specific training sets are a bit smaller than those of Tian et al., as we opted for conservative duplicate and label consistency checks, and we removed 20% of the processed compounds to be used as holdout data for model testing. More specifically, our CYP-specific training sets are composed of 1386 to 1407 compounds, whereas the training sets of Tian et al. are each composed of 1632 compounds (Table S1). As a direct result of the differing data split strategies, our CYP-specific test sets are composed of 343 to 352 compounds, whereas those of Tian et al. are composed of only 104 to 132 compounds. Importantly, the low number of compounds in the test sets of Tian et al. translate to an underrepresentation of substrates that renders them in part insufficient for drawing any robust conclusions: Three of the nine CYPs are represented by fewer than 10 substrates in the test sets of Tian et al. (four substrates in the case of CYP2B6). In contrast, the lowest number of substrates in any of our test sets is 21.

A further point to note is that (disregarding stereochemical information) there is an overlap of a total of 27 compounds between the training and test sets of Tian et al. This could cause an overestimation of model performance, in particular when working with small test sets in connection with the Jaccard score. In the case of the CYP2B6 test set of Tian et al., three out of the four substrates included in this test set are also included as substrates in the training set. In the case of the CYP2C8 test set of Tian et al., the respective number of overlapping substrates (with identical bioactivity labels) is eight out of 12. Given these limitations, we decided to fall back on CV-performance for the direct comparison of both models.

#### 2.6.2. Comparison of the Cross-Validation Performance of the Models

As pointed out above, the models of Tian et al. and our models have been derived from, and tested on, related but not identical data sets. For the sake of comparability with the reports of Tian et al., we referred to the use of the Jaccard score as the primary metric for the quantification of classification performance.

The CYPReact models obtained Jaccard scores ranging from 0.25 to 0.55 during five-fold CV (Table 7). In comparison, our single classifiers obtained consistently better Jaccard scores across the nine CYPs (on average, +0.07), with values ranging from 0.26 to 0.66. Our best models employing the hard voting strategy performed substantially better, with Jaccard scores ranging from 0.33 to 0.79. However, note that these models do not cover the whole space of input molecules (coverage of 0.42 to 0.71 for substrates and of 0.79 to 0.91 for non-substrates).

In terms of compound ranking performance, the CYPReact models and our classifiers built on a single type of descriptors are comparable. The range of AUC values was between 0.83 and 0.92 for the CYPReact models and between 0.80 and 0.92 for our single models. The average AUC for our nine models was 0.02 higher than that of CYPReact, but this difference is not significant.

#### 2.6.3. Comparison of the Applicability Domains

The developers of CYPReact do not provide a definition of the applicability domain of their models. For our models, we show (in Figure 5) that they produce reliable results for compounds that are represented by at least one compound in the training data with a Tanimoto coefficient (based on Morgan2 fingerprints) of 0.7 or higher. Predictions for compounds of interest that are more distantly related to the training data should be treated with additional caution.

## 3. Materials and Methods

### 3.1. Data Sets

#### 3.1.1. Core Data Set

For the purpose of model development, a “core data set” of substrates and non-substrates of nine human CYP isozymes (1A2, 2A6, 2B6, 2C8, 2C9, 2C19, 2D6, 2E1, and 3A4) was compiled from the works of Hunt et al. [32] and Tian et al. [15]. The Hunt data set is a well-curated collection of a total of 484 known substrates of seven of the nine CYPs under investigation (excluding CYPs 2A6 and 2B6). The Tian data set contains a total of 1800 known substrates and non-substrates for the same nine CYPs (Tian et al. provide information on which compounds they used for model training and which ones they used for testing. This information was not considered in this work, because it was found that several CYPs of interest are underrepresented in the test set of Tian et al.—see Table S1 for class label distributions). The substrates included in this data set themselves originate, to a significant part, from the site-of-metabolism data set of Zaretzki et al. [16]. Since the Zaretzki data set has been revised in the meantime [33], the part of the data originating from the original Zaretzki data set was replaced by the data from the revised Zaretzki data set for the purpose of this study.

The exact procedure by which the Hunt and Tian data sets were combined to form the core data set is described in the section “Compilation of the core data set”.

#### 3.1.2. Reference Data Sets for Approved Drugs, Cosmetic Ingredients, and Pesticides

Reference data sets representing the chemical space of approved drugs, cosmetic ingredients, and pesticides were sourced from publicly available databases. The approved drugs data set was acquired from the “approved drugs” subset of the DrugBank database [19,34]. The data set of cosmetic ingredients was obtained from “COSMOS DB” [21] (a database of known cosmetic ingredients compiled by experts from the EU and U.S.) and that of pesticides from the EPA’s Pesticide Chemical Search Database [22], which are both provided by the CompTox Chemicals Dashboard [35]. 

### 3.2. Chemical Data Preprocessing

All data sets were processed as follows (Table 8 provides information on the number of compounds removed during the individual processing steps):

1. Where not provided by the data source (Tian data set), SMILES representations were generated with RDKit [24] using default parameters. Any entries without chemical information were discarded.
2. Molecules for which the SMILES representation could not be parsed by RDKit were discarded.
3. The molecular structures were standardized with the standardize_mol method of the ChEMBL structure pipeline [36,37]. This procedure includes the standardization of unknown chemistry, removal of S group annotations, kekulization, removal of non-explicit H atoms, normalization of frequent substructures, and neutralization. For molecule entries consisting of several connected components or molecules with specified isotopes, the parent molecule was identified with the get_parent_mol method. By this method, all isotope information is removed, known salts and solvents are stripped, duplicate fragments are removed, and the molecule is neutralized.
4. Molecules containing any elements other than H, B, C, N, O, F, Si, P, S, Cl, Se, Br, and I were discarded.
5. Tautomers were canonicalized using the TautomerEnumerator class of RDKit. All stereochemical information was removed during tautomer canonicalization.

#### Processing of Duplicate Molecule Entries

Duplicate data entries were identified based on canonical SMILES representations (without the consideration of stereochemical information). Any duplicate molecules with consistent class labels were merged. Any duplicate molecules with conflicting class labels (i.e., “substrate” and “non-substrate”) were resolved by removing the class labels for the affected CYPs. Any duplicate molecules with incomplete but non-conflicting class labels were resolved by preserving the existing class label value.

### 3.3. Compilation of the Core Data Set

The core data set was compiled from the Hunt and Tian data sets. Both data sets were preprocessed as described in the section “Chemical Data Preprocessing” and combined under the removal of duplicate entries as described in the section “Processing of Duplicate Molecule Entries”. Table 8 provides information on the number of compounds removed during data set combination.

### 3.4. Splitting of the Core Data Set into a Training and a Test Set

For the purpose of model development, the core data set was divided into a training set (80%) and a test set (20%) using the train test split function of scikit-learn [38] with data shuffling and stratification enabled and the random_state parameter set to 42.

### 3.5. Principal Component Analysis

PCA was executed with scikit-learn based on 44 physically meaningful 2D molecular descriptors computed with MOE [30] and selected previously [23]. Prior to performing the PCA, all descriptors were z-score normalized using the StandardScaler class of scikit-learn.

### 3.6. Descriptors

Four types of molecular descriptors were explored in the development of the CYP selectivity models: MACCS keys, Mol2vec descriptors, Morgan fingerprints, and physicochemical descriptors (“RDKit 2D descriptors”). MACCS keys, Morgan fingerprints (radius 2; length 2048 bits), and RDKit descriptors were calculated with RDKit. The set of RDKit 2D descriptors consists of the complete collection of descriptors available in RDKit that do not require a 3D molecular structure for computation. The RDKit 2D descriptors were calculated with default parameters (exception: the Ipc descriptor, for which the “avg” parameter was set to 1 to prevent the generation of values that exceed the definition of the Float32 data type). The Mol2vec descriptors originate from the recently published Mol2vec method [39], which is an unsupervised machine learning approach based on the World2Vec model popular in natural language processing. Mol2vec descriptors were generated by representing molecular substructures as 2048-bit Morgan fingerprints with radius 1 and using a pretrained model based on 19.9 million compounds (provided on GitHub [40]; see Turk et al. [39]) to create a vector representation of the molecules with 300 continuously valued dimensions.

### 3.7. Model Development

#### 3.7.1. General Model Development

For each of the nine CYPs, individual binary classifiers were developed. The machine learning algorithms explored are random forest and support vector machines (with a radial basis function kernel) as implemented in scikit-learn. 

For all models generated with the RF algorithm, the class weight parameter was set to “balanced”, and n_estimators was set to 500. For bootstrapping and feature selection with random forest, a random_state of 42 was used. All SVM models were built with the class weight parameter set to “balanced” and a random_state of 42 for platt scaling (during CV). 

#### 3.7.2. Feature Selection

For use with the RF algorithm, the calculated non-binary features were used as is; for use with the SVM algorithm, they were standardized. Features that showed no variance were deleted from the feature set. Feature selection was carried out using the SelectPercentile class of scikit-learn based on the calculation of the ANOVA-F score for every feature with the f_classif function. 

#### 3.7.3. Cross-Validation and Hyperparameter Optimization

All models were optimized by a grid search performed within a five-fold CV framework (utilizing the StratifiedKFold class of scikit-learn with a random_state of 42 and shuffle set to True). For the RF algorithm, the hyperparameters min_samples_split and max_features were optimized. For the SVM, the hyperparameters C and gamma were optimized. The hyperparameter range searched during the grid search is reported in Table 9.

### 3.8. Analysis of Feature Importance

Feature importance of individual MACCS keys and RDKit 2D descriptors was assessed with the random forest algorithm as Gini importance [41]. This value is calculated as the weighted decrease of Gini impurity introduced by a feature averaged over all decision trees. Finally, the values of all features are normalized in order to obtain the relative importance for each feature. The Gini importance was obtained from the feature_importance attribute of an RF classifier trained on either MACCS keys or the RDKit 2D descriptors.

### 3.9. Performance Metrics

The MCC was employed as the primary metric for measuring the classification performance of the models. The MCC is a balanced metric, where −1 is the lower bound (indicating total disagreement between prediction and ground truth) and 1 is the upper bound (indicating perfect classification). In order to enable more direct comparisons of this work and that of others, the Jaccard score and the AUC were computed. Note that for consensus classifiers using the majority vote strategy, the AUC cannot be calculated, as these classifiers do not provide a probability estimation of class membership.

## 4. Conclusions

Based on an extended set of substrates and non-substrates of nine CYPs of relevance to xenobiotic metabolism, we developed a battery of single and combined classifiers based on different machine learning algorithms and descriptors. On holdout data, the best single models yielded MCCs between 0.32 and 0.70, and the best combined models (all hard voting models) achieved MCCs between 0.37 and 0.81, depending on the CYP isozyme.

For the hard voting models, the coverage is reduced for the benefit of better prediction accuracy. Use of these models is recommended when the correctness of predictions is a priority over coverage. For example, the prediction of a compound of interest by hard voting classifiers as a non-substrate can add confidence that the compound is not a CYP substrate. In contrast, the single classifiers will be preferred in cases where maximum coverage is a priority.

Our recommendation is to use both types of models in parallel, as this strategy will provide additional information that can prove valuable for the interpretation of predictions and for deciding on what compounds to follow up on.

The applicability domain of the individual classifiers should be closely observed. We have shown that the classifiers perform well in particular on compounds that have a structurally related neighbor in the training data (i.e., at least one training instance with a Tanimoto coefficient, based on Morgan2 fingerprints, of 0.7 or higher). Predictions for compounds of interest that are more distantly related to the training data should be treated with additional caution.

All of the best single classifiers and hard voting classifiers presented in this work are accessible via the “CYPstrate” module of the New E-Resource for Drug Discovery (NERDD) [42] at https://nerdd.univie.ac.at/cypstrate/, accessed on 25 July 2021. The web service meets modern security standards and allows users to immediately delete all their data. The web service reports, upon request, the nearest training instances so that users can make an informed decision about whether or not to trust a prediction.

## Figures and Tables

**Table 1:** Distribution of substrates and non-substrates in the core data set in comparison to a version that does not make use of the data of Hunt et al. [13].

	Number of Substrates			Number of Non-Substrates		
CYP	Without Hunt et al.	With Hunt et al. (This Is the Core Data Set)	Difference in %	Without Hunt et al.	With Hunt et al. (This Is the Core Data Set)	Difference in % ^1^
1A2	280	296	+5.4	1441	1428	−0.9
2A6	107	107	0.0	1607	1607	0.0
2B6	150	150	0.0	1561	1561	0.0
2C8	140	149	+6.0	1572	1565	−0.5
2C9	237	253	+6.3	1484	1469	−1.0
2C19	225	242	+7.0	1493	1481	−0.8
2D6	276	304	+9.2	1441	1425	−1.1
2E1	148	156	+5.1	1566	1556	−0.6
3A4	478	520	+8.1	1241	1239	−0.2

^1^ In some cases, the merging of the data from Tian et al. and Hunt et al. did in fact lead to a reduction in the number of non-substrates. This is because of inconsistencies identified in the bioactivity labels of compounds present in both data sets. Any compounds with conflicting activity labels were rejected during data processing (see Section 3 Materials and Methods for details).

**Table 2:** Performance of the best-performing classifiers by MCC built on a single feature set.

CYP		1A2	2A6	2B6	2C8	2C9	2C19	2D6	2E1	3A4
**Algorithm ^1^**		RF	RF	RF	RF	SVM	RF	SVM	SVM	SVM
**Descriptors ^2^**		MFP	R2D	MFP	MK	M2V	MK	R2D	MFP	R2D
**Performance during CV ^3^**	**MCC**	0.58	0.44	0.44	0.36	0.49	0.49	0.59	0.56	0.71
	**Jaccard**	0.48	0.31	0.32	0.26	0.38	0.39	0.49	0.42	0.66
	**AUC**	0.88	0.89	0.86	0.80	0.87	0.86	0.92	0.88	0.92
**Performance on the test set**	**MCC**	0.50	0.32	0.33	0.40	0.48	0.59	0.61	0.40	0.70
	**Jaccard**	0.41	0.22	0.24	0.29	0.37	0.48	0.50	0.28	0.65
	**AUC**	0.88	0.85	0.85	0.90	0.89	0.91	0.92	0.80	0.90

^1^ Algorithm with which the best model was obtained for a particular CYP isozyme. ^2^ Descriptor set with which the best model was obtained for a particular CYP isozyme: MFP, Morgan2 fingerprints; R2D, RDKit 2D descriptors; MK, MACCS Keys; M2V, Mol2vec. ^3^ Median over the five folds of CV.

**Table 3:** Overview of the composition of the combined models.

No. Classifiers Underlying a Combined Model	Feature Sets	Classification Algorithms	Voting Strategy	No. Combinations (i.e., Number of Consensus Models)
2	MACCS keys Morgan2 fingerprints RDKit 2D descriptors Mol2vec descriptors	RF, SVM	soft max hard (min consensus = 2)	72 ^1^
3			soft max hard (min consensus = 3)	96 ^1^
4			soft max hard (min consensus = 3) hard (min consensus = 4)	64 ^1^

^1^ Resulting from the combination of the number of ways to select two/three/four out of four feature sets, with two machine learning algorithms for each feature set, and with three/four voting modes. For the combination of two classifiers to a consensus model, the number of combinations (72) results from 6 × 4 × 3 possibilities.

**Table 4:** Performance of the best-performing soft voting classifiers by MCC.

CYP		1A2	2A6	2B6	2C8	2C9	2C19	2D6	2E1	3A4
**Model Setup ^1^**		RF MFP + RF MK	RF R2D + RF MK	RF R2D + RF MFP	RF R2D + RF M2V	RF R2D + RF MFP + SVM MK + RF M2V	RF R2D + SVM MFP	RF R2D + RF MFP + RF MK + SVM M2V	RF R2D + SVM MFP + RF MK + SVM M2V	SVM R2D + RF MK + SVM M2V
**Performance during CV (Median over the 5 Folds of CV)**	**MCC**	0.58	0.47	0.46	0.33	0.50	0.55	0.61	0.62	0.72
	**Jaccard**	0.48	0.32	0.33	0.24	0.40	0.40	0.51	0.44	0.67
	**AUC**	0.90	0.89	0.88	0.79	0.87	0.88	0.93	0.89	0.94
**Performance on the Test Set**	**MCC**	0.58	0.39	0.36	0.38	0.50	0.54	0.58	0.46	0.70
	**Jaccard**	0.49	0.26	0.26	0.28	0.41	0.41	0.47	0.32	0.65
	**AUC**	0.91	0.84	0.86	0.87	0.90	0.90	0.92	0.89	0.91

^1^ Abbreviations of descriptor (sets): MFP, Morgan2 fingerprints; R2D, RDKit 2D descriptors; MK, MACCS Keys; M2V, Mol2vec.

**Table 5:** Performance of the best-performing hard voting classifiers by MCC.

CYP		1A2	2A6	2B6	2C8	2C9	2C19	2D6	2E1	3A4
**Model Setup ^1^**		RF R2D + RF MFP + RF MK + RF M2V	RF R2D + RF MK + RF M2V	RF R2D + RF MFP + RF MK + RF M2V	RF MFP + RF MK	RF R2D + SVM MFP + RF MK + RF M2V	RF R2D + SVM MFP + RF MK + RF M2V	RF R2D + RF MFP + SVM MK + RF M2V	RF R2D + RF MK + SVM M2V	RF R2D + SVM MFP + RF MK + SVM M2V
**Minimum Consensus ^2^**		4	3	4	2	4	4	4	3	4
**Performance during CV (Median over the 5 Folds of CV)**	**MCC**	0.76	0.55	0.62	0.45	0.6	0.66	0.81	0.63	0.85
	**Jaccard**	0.66	0.39	0.48	0.33	0.44	0.52	0.72	0.45	0.79
	**Coverage of Substrate**	0.52	0.71	0.62	0.67	0.42	0.54	0.54	0.56	0.66
	**Coverage of Non-Substrate**	0.82	0.91	0.79	0.88	0.79	0.81	0.84	0.93	0.85
**Performance on the Test Set**	**MCC**	0.81	0.37	0.54	0.49	0.64	0.72	0.72	0.58	0.81
	**Jaccard**	0.72	0.25	0.38	0.35	0.5	0.58	0.61	0.42	0.75
	**Coverage of substrate**	0.56	0.62	0.5	0.7	0.39	0.4	0.52	0.68	0.72
	**Coverage of Non-Substrate**	0.82	0.9	0.83	0.9	0.8	0.81	0.85	0.91	0.92

^1^ Abbreviations of descriptor (sets): MFP, Morgan2 fingerprints; R2D, RDKit 2D descriptors; MK, MACCS Keys; M2V, Mol2vec. ^2^ Minimum number of underlying classifiers required to agree in order to produce a prediction.

**Table 6:** Performance of the best-performing max voting classifiers by MCC.

CYP		1A2	2A6	2B6	2C8	2C9	2C19	2D6	2E1	3A4
**Model Setup ^1^**		RF R2D + SVM MFP + RF MK + SVM M2V	RF R2D + RF MK	RF R2D + RF MFP	RF R2D + RF M2V	RF R2D + SVM MFP + SVM MK + RF M2V	RF R2D + SVM MFP	RF R2D + SVM MK + RF M2V	SVM R2D + RF MFP + RF MK + SVM M2V	RF R2D + SVM MK + RF M2V
**Performance during CV (Median over the 5 Folds of CV)**	**MCC**	0.58	0.47	0.46	0.33	0.47	0.55	0.60	0.61	0.71
	**Jaccard**	0.45	0.32	0.33	0.24	0.37	0.40	0.49	0.42	0.66
	**AUC**	0.90	0.88	0.88	0.79	0.86	0.88	0.91	0.88	0.92
**Performance on the Test Set**	**MCC**	0.70	0.39	0.36	0.38	0.54	0.54	0.56	0.46	0.71
	**Jaccard**	0.58	0.26	0.26	0.28	0.43	0.41	0.45	0.32	0.66
	**AUC**	0.92	0.83	0.86	0.87	0.90	0.90	0.90	0.88	0.92

^1^ Abbreviations of descriptor (sets): MFP, Morgan2 fingerprints; R2D, RDKit 2D descriptors; MK, MACCS Keys; M2V, Mol2vec.

**Table 7:** Comparison of the performance of the CYPReact models and our best classifiers.

CYP	Single Classifier				Hard Voting Classifier			CypReact	
	Algorithm ^1^	Descriptors ^2^	Jaccard Score	AUC	Model Setup	Minimum Consensus ^3^	Jaccard Score	Jaccard Score	AUC
1A2	RF	Morgan FP	0.48	0.88	RF RDKit 2D + RF Morgan FP + RF MACCS keys + RF Mol2Vec	4	0.66	0.39	0.86
2A6	RF	RDKit 2D	0.31	0.89	RF RDKit 2D + RF MACCS keys + RF Mol2Vec	3	0.39	0.28	0.84
2B6	RF	Morgan FP	0.32	0.86	RF RDKit 2D + RF Morgan FP + RF MACCS keys + RF Mol2Vec	4	0.48	0.28	0.86
2C8	RF	MACCS keys	0.26	0.80	RF Morgan FP + RF MACCS keys	2	0.33	0.25	0.84
2C9	SVM	Mol2Vec	0.38	0.87	RF RDKit 2D + SVM Morgan FP + RF MACCS keys + RF Mol2Vec	4	0.44	0.30	0.83
2C19	RF	MACCS keys	0.39	0.86	RF RDKit 2D + SVM Morgan FP + RF MACCS keys + RF Mol2Vec	4	0.52	0.30	0.83
2D6	SVM	RDKit 2D	0.49	0.92	RF RDKit 2D + RF Morgan FP + SVM MACCS keys + RF Mol2Vec	4	0.72	0.40	0.87
2E1	SVM	Morgan FP	0.42	0.88	RF RDKit 2D + RF MACCS keys + SVM Mol2Vec	3	0.45	0.30	0.87
3A4	SVM	RDKit 2D	0.66	0.92	RF RDKit 2D + SVM Morgan FP + RF MACCS keys + SVM Mol2Vec	4	0.79	0.55	0.92

^1^ Algorithm with which the best model was obtained for a particular isozyme. ^2^ Descriptor set with which the best model was obtained for a particular isozyme. ^3^ Minimum number of underlying classifiers required to agree in order to produce a prediction.

**Table 8:** Number of molecules removed during each processing step.

Number of Compounds	Core Data Set	COSMOS DB	EPAPCS	DrugBank
**Forming the unprocessed data set**	2284 ^1^	7035	4035	2506
**Removed due to missing SMILES**	0	1618	751	0
**Removed due to unparsable SMILES**	0	23	43	6
**Removed by the element filter**	0	392	344	208
**Removed by the duplicate filter**	453	522	369	74
**Forming the processed data set**	1831	4480	2528	2218

^1^ Sum of the number of compounds included in the Hunt and Tian data sets.

**Table 9:** Hyperparameter ranges explored by grid search during model optimization.

Algorithm	Parameter	Explored Values
RF	min_samples_split	2, 4, 8, 16, 32, 64, 128
	max_features	0.05, 0.1, 0.2, 0.4, 0.8, sqrt
SVM	C	1 × 10^−2^, 1 × 10^−1^, 1 × 10^0^, 1 × 10^1^, 1 × 10^2^, 1 × 10^3^
	gamma	1, 1 × 10^−1^, 1 × 10^−2^, 1 × 10^−3^, 1 × 10^−4^, 1 × 10^−5^
SelectPercentile	percentile	10, 40, 70, 100

## Data Availability

All data utilized in this work are available from the respective references as indicated in the manuscript text. The best models presented in this work are accessible free of charge via the “CYPstrate” module of NERDD at https://nerdd.univie.ac.at/cypstrate/, accessed on 25 July 2021.

## Supplementary Materials

The following are available online: Figure S1: MCCs (median over the 5 folds of the CV) plotted against the number of substrates in a training set, for all 72 single classifiers generated for the nine CYP isozymes by the combination of two machine learning algorithms and four descriptor sets; Table S1: Distribution of substrates and non-substrates per isozyme and data set; Table S2: RDKit 2D descriptors ranked by at least one RF classifier among the five most important features; all data used for model training and testing.

## Sample Availability

Not available.
