# Supplementary material for: CYPstrate: A Set of Machine Learning Models for the Accurate Classification of Cytochrome P450 Enzyme Substrates and Non-Substrates
Source: Molecules. 2021 Aug 2;26(15):4678. doi: 10.3390/molecules26154678 (PMC8347321; doi:10.3390/molecules26154678)
Supplement: Supplementary file 1 [file molecules-26-04678-s001.zip › molecules-1317149-supplementary.pdf]

*Supporting Information*

# CYPstrate: A set of machine learning models for the accurate classification of cytochrome P450 enzyme substrates and non-substrates

**Malte Holmer [1], Christina de Bruyn Kops [1], Conrad Stork [1] and Johannes Kirchmair [1,2*]**

[1] Center for Bioinformatics (ZBH), Department of Informatics, Universität Hamburg, 20146 Hamburg, Germany; malte.holmer@studium.uni-hamburg.de (M.H.); kops@zbh.uni-hamburg.de (C.d.B.K.); stork@zbh.uni-hamburg.de (C.S.)

[2] Division of Pharmaceutical Chemistry, Department of Pharmaceutical Sciences, University of Vienna, 1090 Vienna, Austria.

* Correspondence: johannes.kirchmair@univie.ac.at

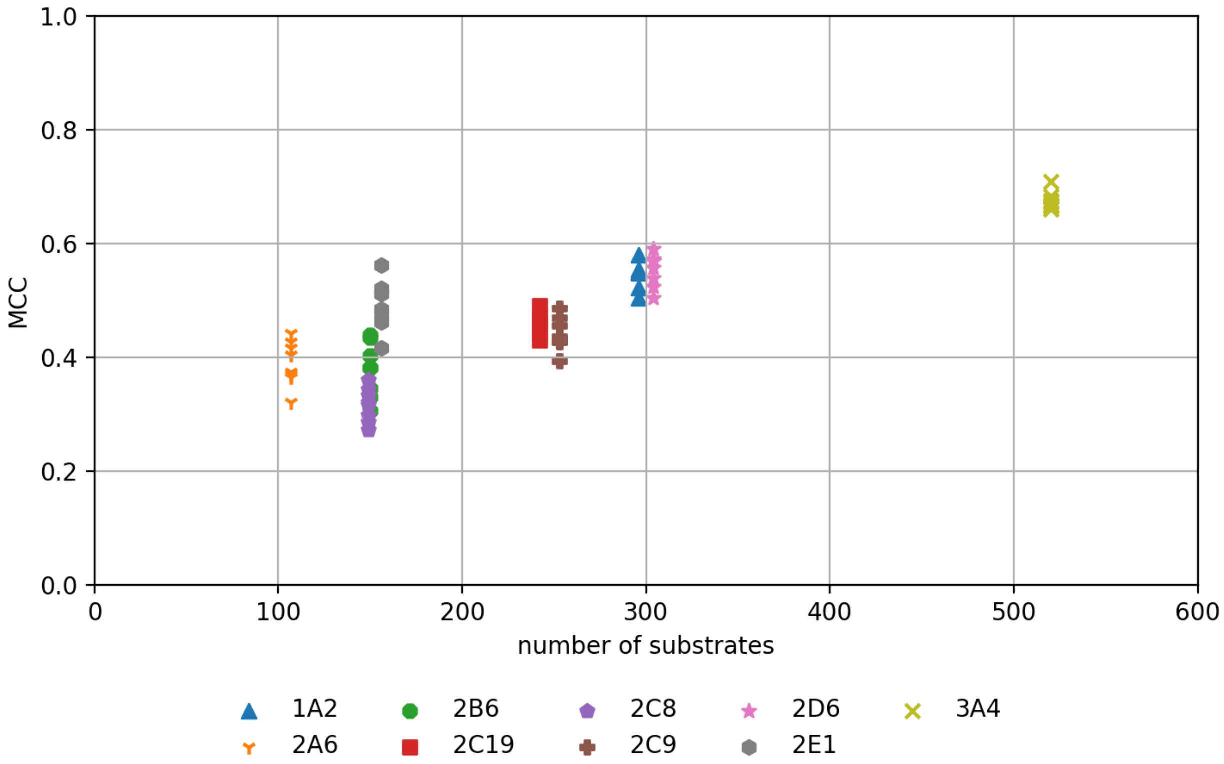


**Figure S1:** MCCs (median over the 5 folds of the CV) plotted against the number of substrates in a training set, for all 72 single classifiers generated for the nine CYP isozymes by the combination of two machine learning algorithms and four descriptor sets.

**Table S1.** Distribution of substrates and non-substrates per CYP isozyme and data set.

| CYP | Class | Tian et al. training set[1] | Tian et al. test set[1] | Hunt et al. data set[1] | Core data set | Core data set (training) | Core data set (test) |
|---|---|---|---|---|---|---|---|
| 1A2 | non-substrate | 1361 | 100 | - | 1428 | 1142 | 286 |
| | substrate | 271 | 24 | 201 | 296 | 237 | 59 |
| 2A6 | non-substrate | 1527 | 100 | - | 1607 | 1285 | 322 |
| | substrate | 105 | 6 | - | 107 | 86 | 21 |
| 2B6 | non-substrate | 1481 | 100 | - | 1561 | 1248 | 313 |
| | substrate | 151 | 4 | - | 150 | 120 | 30 |
| 2C8 | non-substrate | 1490 | 100 | - | 1565 | 1252 | 313 |
| | substrate | 142 | 12 | 107 | 149 | 119 | 30 |
| 2C9 | non-substrate | 1406 | 100 | - | 1469 | 1175 | 294 |
| | substrate | 226 | 28 | 194 | 253 | 202 | 51 |
| 2C19 | non-substrate | 1414 | 100 | - | 1481 | 1184 | 297 |
| | substrate | 218 | 20 | 184 | 242 | 194 | 48 |
| 2D6 | non-substrate | 1362 | 100 | - | 1425 | 1140 | 285 |
| | substrate | 270 | 21 | 203 | 304 | 243 | 61 |
| 2E1 | non-substrate | 1487 | 100 | - | 1556 | 1244 | 312 |
| | substrate | 145 | 6 | 106 | 156 | 125 | 31 |
| 3A4 | non-substrate | 1157 | 100 | - | 1239 | 991 | 248 |
| | substrate | 475 | 32 | 304 | 520 | 416 | 104 |

[1] Class label distributions were analyzed prior to the processing of the data.

**Table S2. RDKit 2D descriptors ranked by at least one RF classifier among the five most important features.**

| Descriptor name | Description |
|---|---|
| **EState_VSA10** | Approximated van der Waals surface area of all atoms with an EState value [31] within a given interval. |
| **EState_VSA2** | |
| **FractionCSP3** | Fraction of C atoms that are sp3 hybridized. |
| **Kappa1** | First kappa index characterizing aspects of the molecular shape by comparing a molecule to its possible extreme shapes [42]. |
| **MinPartialCharge** | Minimum partial charge of an atom calculated by the iterative procedure [43]. |
| **MolLogP** | Log of the octanol/water partition coefficient calculated by an atomic contribution model [44]. |
| **NumAromaticCarbocycles** | Number of aromatic carbocycles for a molecule. |
| **NumAromaticRings** | Number of aromatic rings for a molecule. |
| **NumHDonors** | Number of Hydrogen Bond Donors |
| **PEOE_VSA1** | Approximated van der Waals surface area of all atoms with a partial charge within a given interval. The partial charge is calculated by the Partial Equalization of Orbital Electronegativities (PEOE) method [43]. |
| **SMR_VSA1** | Approximated van der Waals surface area of all atoms with a molecular refractivity within a given interval (-inf < x < 1.29). Molecular refractivity is calculated as reported in Ref [44]. |
| **SMR_VSA5** | |
| **SMR_VSA6** | |
| **SMR_VSA7** | |
| **SlogP_VSA3** | Approximated van der Waals surface area of all atoms with a logP within a given interval. Log of the octanol/water partition coefficient (logP) is calculated as reported in Refs [44,45]. |
| **SlogP_VSA5** | |
| **TPSA** | Implementation of the topological polar surface area descriptor of Ertl et al. [45]. |
| **VSA_EState10** | Approximated van der Waals surface area of all atoms with an EState value within a given interval. |

| | |
|---|---|
| **VSA_EState3** | |
| **VSA_EState6** | |
| **fr_Al_OH** | Number of aliphatic hydroxyl groups. |
| **fr_NH0** | Number of tertiary amines. |
| **fr_benzene** | Number of benzene rings. |
| **qed** | Weighted quantitative estimate of drug-likeness ($QED_w$) [46]. |
